# Supplementary material for: Hierarchically structured lithium titanate for ultrafast charging in long-life high capacity batteries
Source: Nat Commun. 2017 May 26;8:15636. doi: 10.1038/ncomms15636 (PMC5458554; doi:10.1038/ncomms15636)
Supplement: Supplementary Information — Supplementary Figures and Supplementary Table [file ncomms15636-s1.pdf]

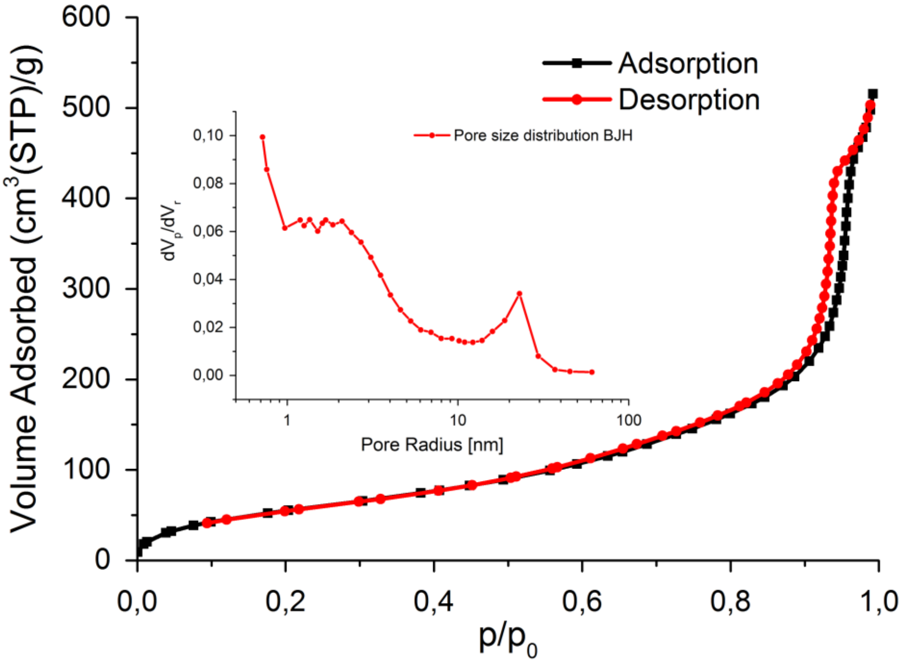

Supplementary Figure 1 Nitrogen adsorption/desorption isotherms and Barrett-Joyner-Halenda (BJH) pore size distribution (insert) analysis for the as-obtained LTO.

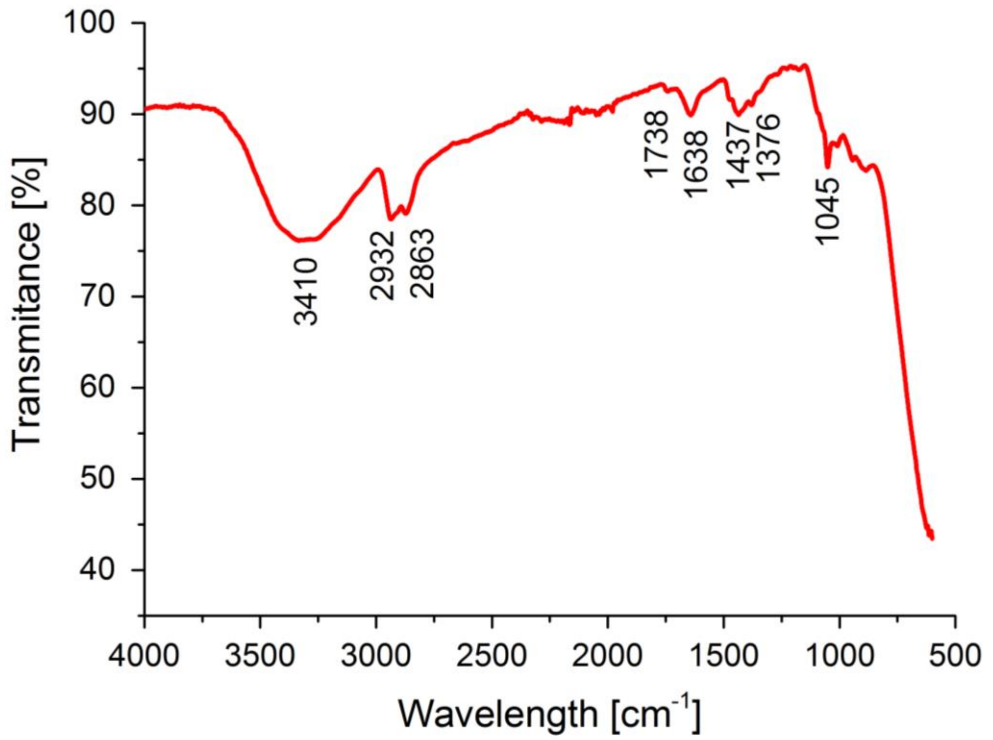

Supplementary Figure 2 Fourier transform infrared (FT-IR) spectroscopy of obtained LTO. The surface of NPs was examined by means of FTIR. Broad and strong peak with the maximum at 3410 cm^{-1} and peak at 1638 cm^{-1} correspond to O-H groups at the surface of NPs and adsorbed water. The 2932 cm^{-1} and 2863 cm^{-1} peaks are due to the asymmetric and symmetric stretching of the methylene groups from butoxy groups and 1,4-BD. Then it was confirmed by the peaks located at 1437, 1376 cm^{-1} (wagging modes of methylene groups) and 1045 cm^{-1} which is due to stretching of C-O bonds. Peak at 1738 cm^{-1} is probably due to some carboxylic group from lithium acetate and it corresponds to C=O stretching. As the conclusion, nanoparticles are mostly covered with 1,4-butanediol and butoxy groups, which are not completely removed during washing step. This shows that 1,4-BD serve as capping agents preventing further growth of particles.

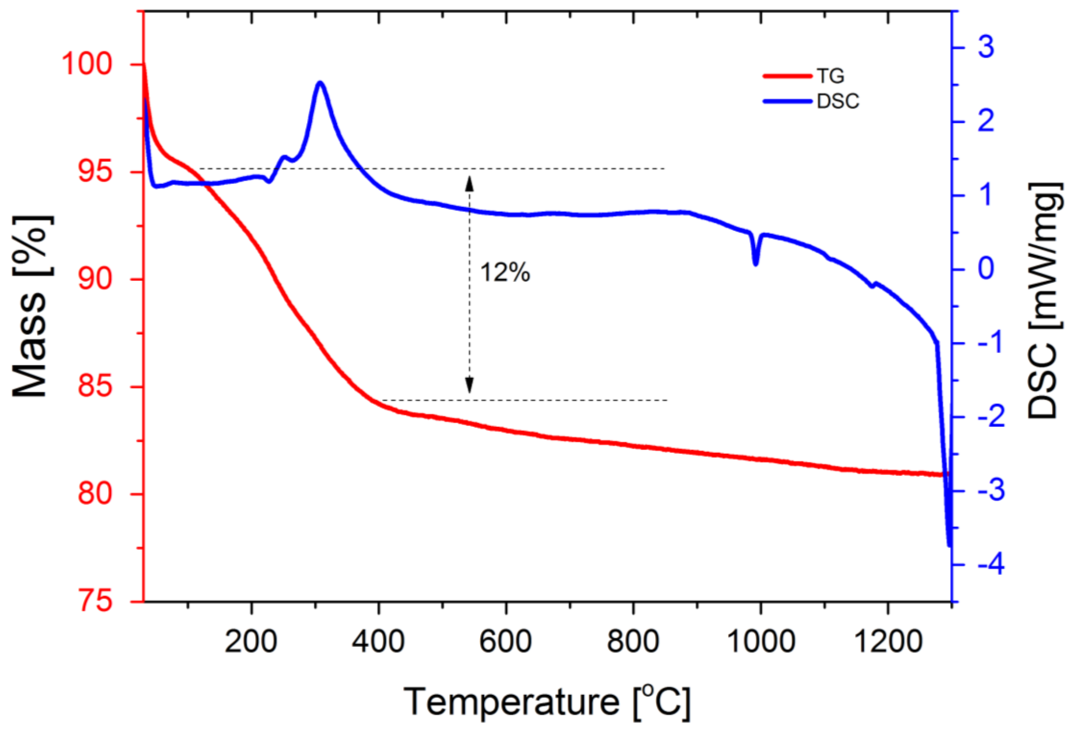

Supplementary Figure 3 Thermal gravimetric analysis and differential scanning calorimetry of as-obtained LTO. The amount of adsorbed organics was evaluated by thermal analysis. Up to around 120oC, adsorbed water is removed from the nanoparticles. Then gradually –OH groups and organic species are removed from the surface (exothermic peaks at 251 and 307°C).

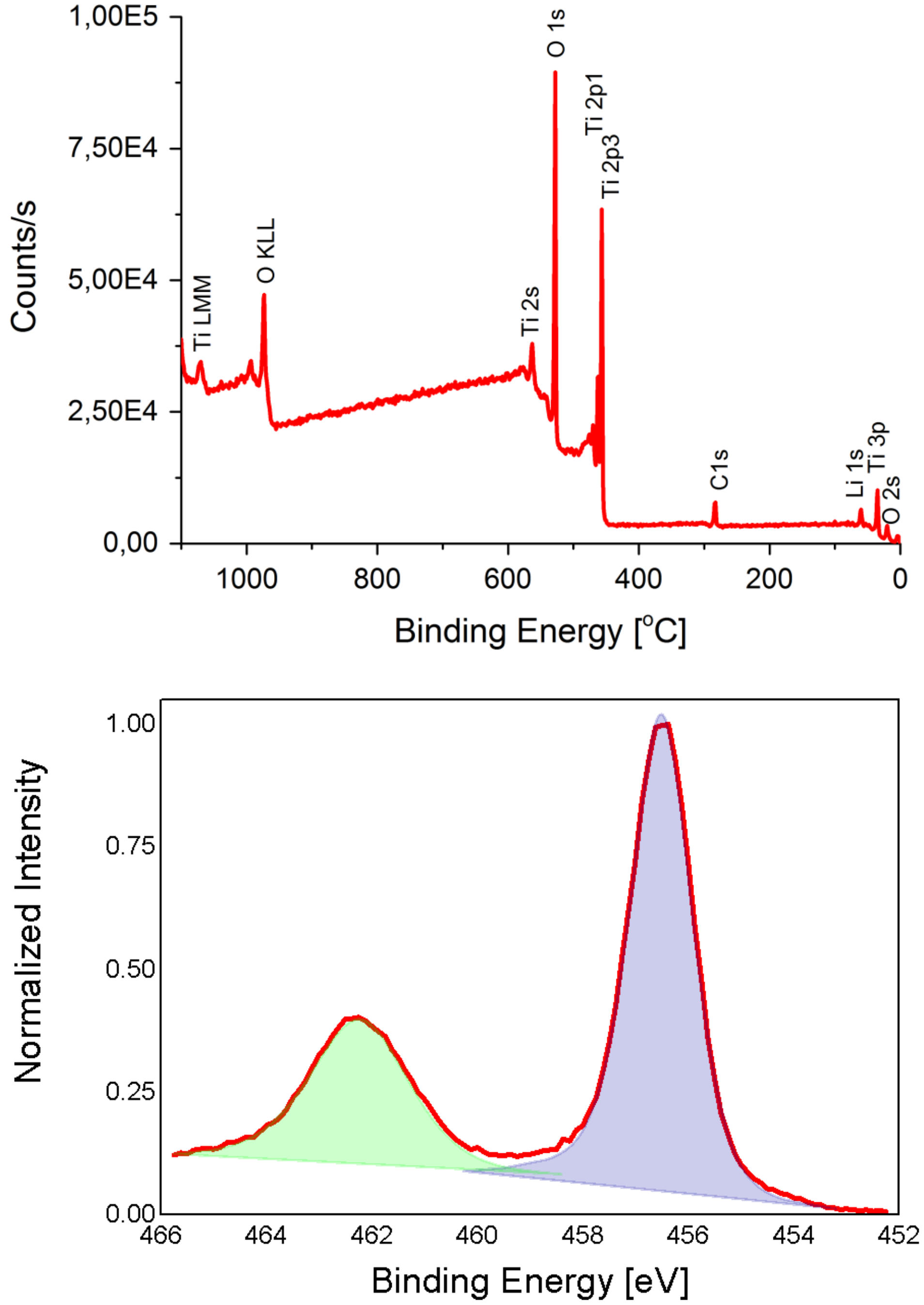

Supplementary Figure 4. XPS spectra **(upper)** and Ti2p XPS spectra **(bottom)** of hierarchically nanostructured LTO.

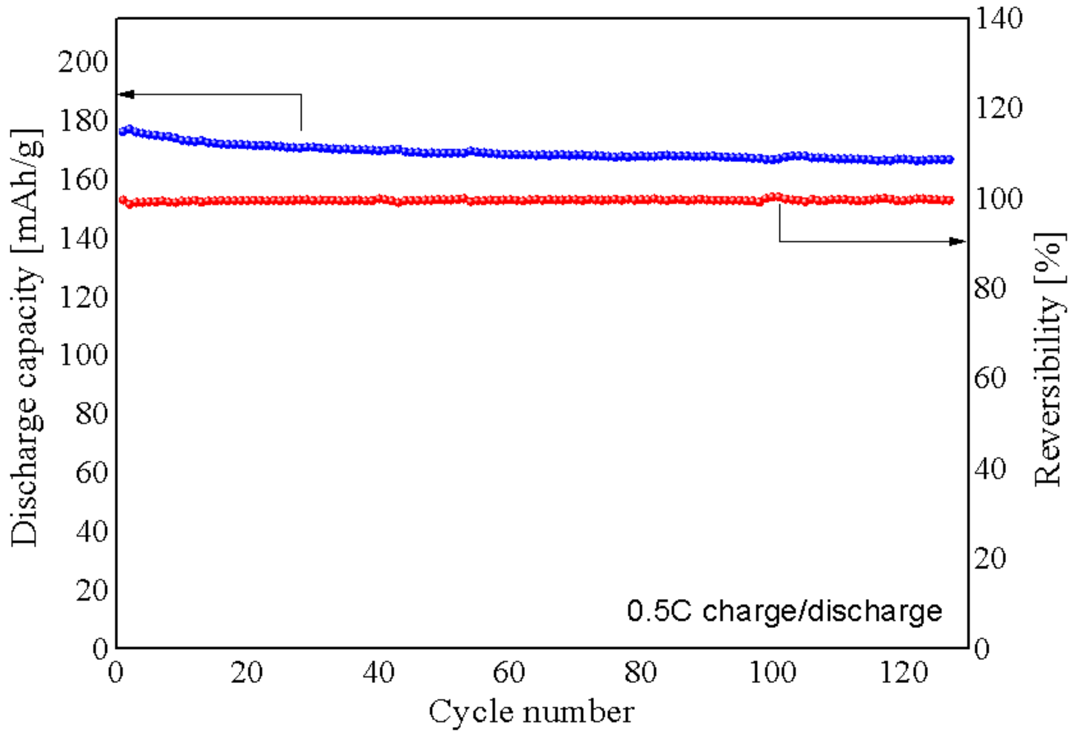

Supplementary Figure 5. Initial 120 cycles of $Li/Li^{+}/LTO$ battery at 0,5 C current rate.

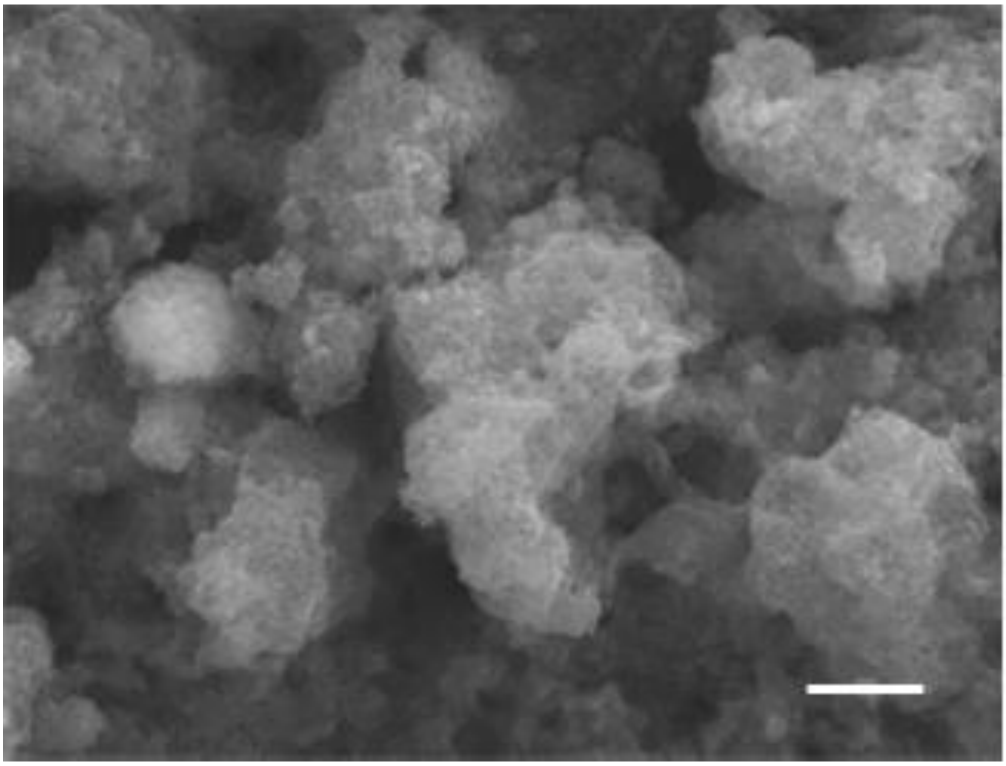

Supplementary Figure 6. SEM picture of anode layer prepared from LTO and cycled 300 times at 50C. Scale bar, 1µm.

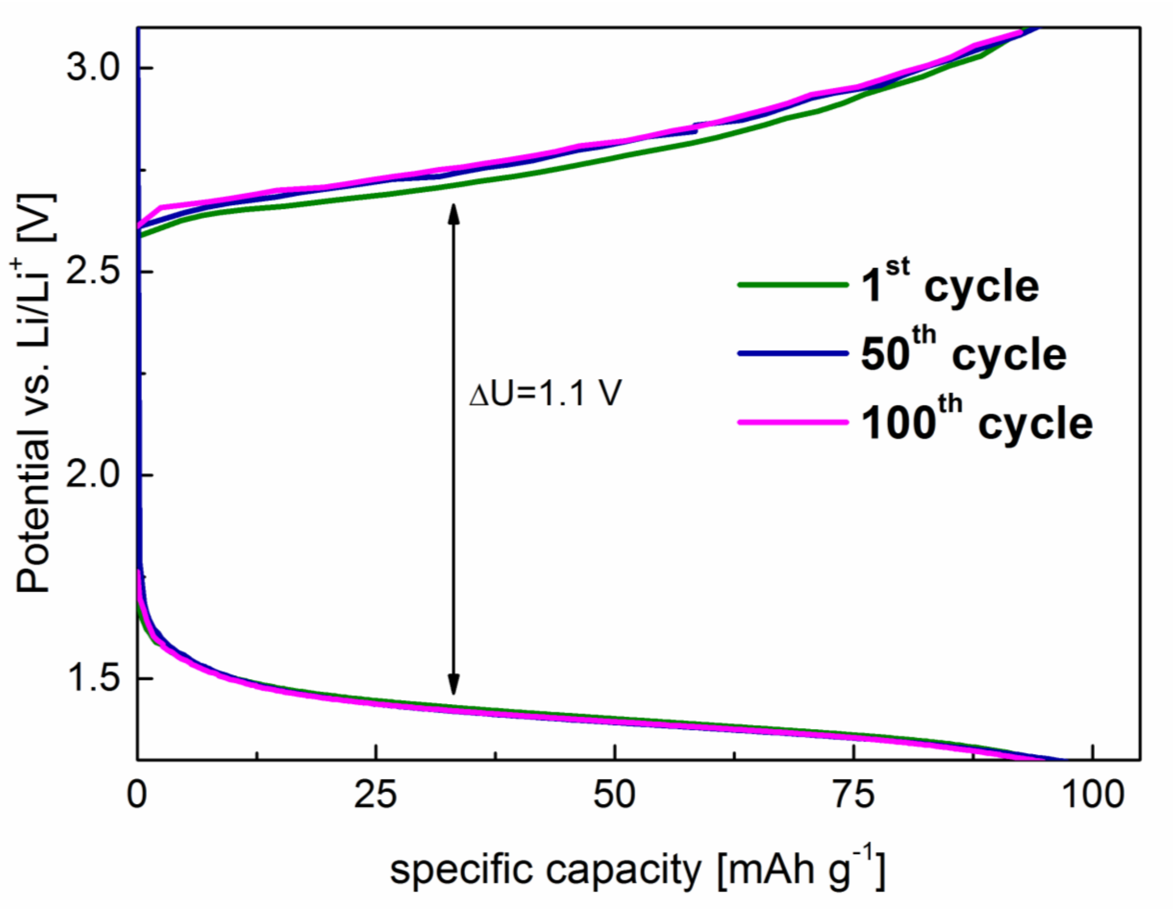

Supplementary Figure 7. Selected charge/discharge profiles for Li/Li^+/LTO battery charged at 500 C and discharged at 50 C current rate.

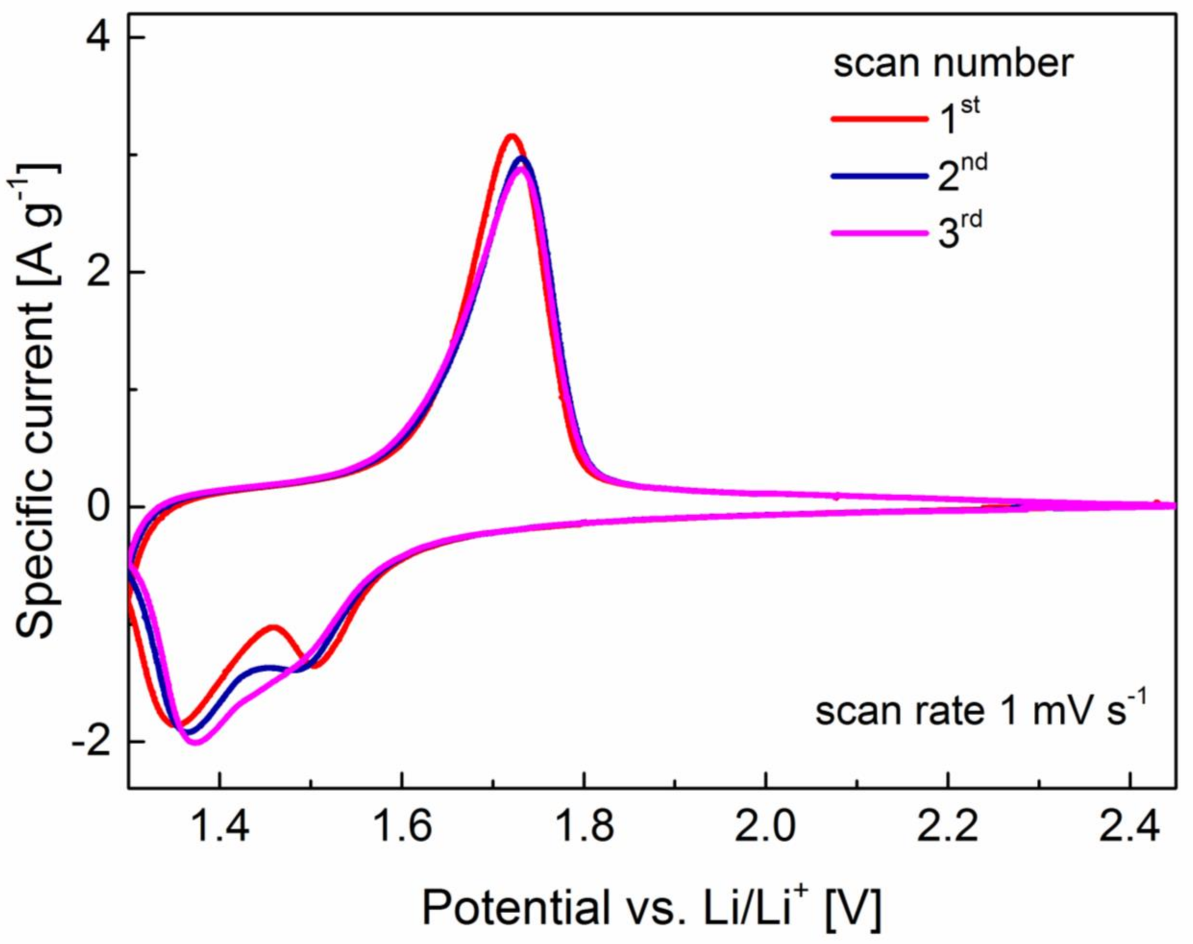

Supplementary Figure 8. Results of cycling voltammetry studies of $Li/Li^{+}/LTO$ battery performed with 1 mV s^{-1} scanning rate.

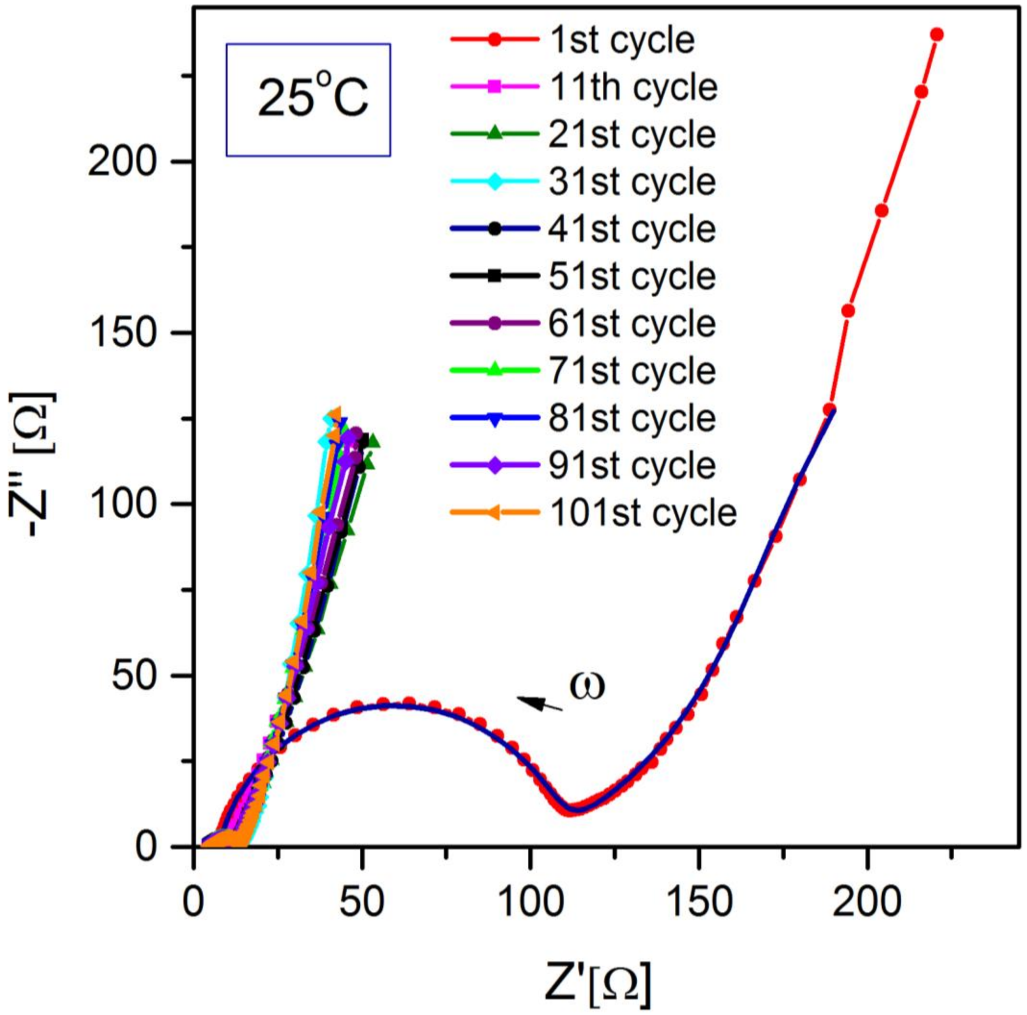

Supplementary Figure 9. EIS data on cycling, recorded for Li/Li$^+$/LTO cell.

Supplementary Table 1 Atomic composition of as-obtained LTO calculated from XPS results.

	C	O	Ti	Li	Li/Ti	O/(Li+Ti)
**LTO 1st**	11.7	58.0	20.4	9.9	0.49	1.91
**LTO 2nd**	10.7	58.2	20.8	10.3	0.50	1.87
